# A ‘NanoSuit’ successfully protects petals of cherry blossoms in high vacuum: examination of living plants in an FE-SEM

**DOI:** 10.1038/s41598-018-19968-w

**Published:** 2018-01-26

**Authors:** Sayuri Takehara, Yasuharu Takaku, Hiroshi Suzuki, Isao Ohta, Masatsugu Shimomura, Takahiko Hariyama

**Affiliations:** 1 Department of Biology, Hamamatsu University School of Medicine, 1-20-1 Handayama, Higashi-ku, Hamamatsu 431-3192 Japan; 2 Department of Chemistry, Hamamatsu University School of Medicine, 1-20-1 Handayama, Higashi-ku, Hamamatsu 431-3192 Japan; 3 Laboratory for Ultrastructure Research, Research Equipment Center, Hamamatsu University School of Medicine, 1-20-1 Handayama, Higashi-ku, Hamamatsu 431-3192 Japan; 4 Department of Bio- and Material Photonics, Chitose Institute of Science and Technology, 758-65 Chitose, Hokkaido, 066-8655 Japan

## Abstract

Land plants have evolved on dry land and developed surface barriers to protect themselves from environmental stresses. We have previously reported that polymerization of a natural extracellular substance (ECS) on the outer surface of animals by electron beam or plasma irradiation, can give rise to a nano-scale layer, termed the “NanoSuit”, which can keep small animals alive under the high vacuum of a scanning electron microscope (SEM). In the present research, we have focused on plants, using petals of cherry blossoms, as experimental specimens and examined their behavior under high vacuum conditions. Experiments on healthy living petals have demonstrated that without any pre-treatment, the overall morphology of specimens is well preserved and intact after imaging in an SEM, suggesting that natural substances on the petal surface behave like animal ECS and form a NanoSuit following irradiation with an electron beam. Furthermore, we have shown that the surface material can be extracted with chloroform and polymerized into a free-standing membrane by plasma irradiation. From our results, we conclude that surface materials, which have the ability to prevent water loss under natural conditions, increase the barrier ability and can protect plants under high vacuum conditions.

## Introduction

Land plants have evolved on dry land and developed surface barriers to protect themselves from desiccation, UV exposure, high irradiation, and other abiotic environmental stresses^1–3^. The unique surface microstructures on plants have been studied by scanning electron microscope (SEM)^4–6^. Plant surfaces also exhibit a hydrophobic cuticle, which is composed of cutin and cuticular waxes^3,4,7^. Such cuticular material has been documented for nearly all plant surfaces including petals, stems and leaves.

 Field emission scanning electron microscopes (FE-SEMs) are instruments for observing surface fine structure at high resolution. It is, however, necessary to evacuate the specimen chamber to a high vacuum level (10^−3^–10^−6^ Pa) to prevent scattering by molecules in the air. Since living organisms including plants contain approximately 70 to 80% water, it has been thought necessary to pre-treat such biological specimens to achieve minimal tissue damage and maximal preservation of the structure for SEM observations. The treatments include chemical fixation prior to dehydration, freeze-drying or critical point drying, and metal coating (conventional methods)^8^. These complex procedures, however, preclude the direct observation of living tissues. Furthermore, even in fixed specimens, the evaporation of solution from samples often produces unwanted structural changes.

We have shown that living organisms covered with either a layer of natural extracellular substance (ECS) or an artificial substance mimicking the ECS and polymerized by electron beam irradiation or plasma irradiation, become highly tolerant to the high vacuum in an SEM. The polymerized layer, which we call a “NanoSuit”, can protect the organism during observation in an SEM^9–12^. We have recently improved the technique to maintain wet tissues freshly excised from intact animals or cultured cells with a Surface Shield Enhancer (SSE) solution that consists of glycerin and electrolytes and find that the fine structure of the SSE treated specimens is superior to that of conventionally prepared specimens^13^.

These investigations of the NanoSuit have been carried out mainly on animal specimens. However, it is still unknown whether our new technique could form a similar barrier to desiccation in plants. In the present investigations, we use the high vacuum conditions in the observation chamber of the FE-SEM as the experimental space and examine the response of flower petals, which are fragile compared to other parts, e.g. stems and leaves, to the high vacuum conditions. We show that natural surface substances on petals can be used to protect them from desiccation under high vacuum, and once the substance turns into the NanoSuit, the barrier ability increases. As a consequence, direct observations of living petals in the hydrated state can be performed with high resolution in an FE-SEM.

## Results

### The plant “NanoSuit” plays a significant role as a barrier preventing desiccation

**Figure 1:**
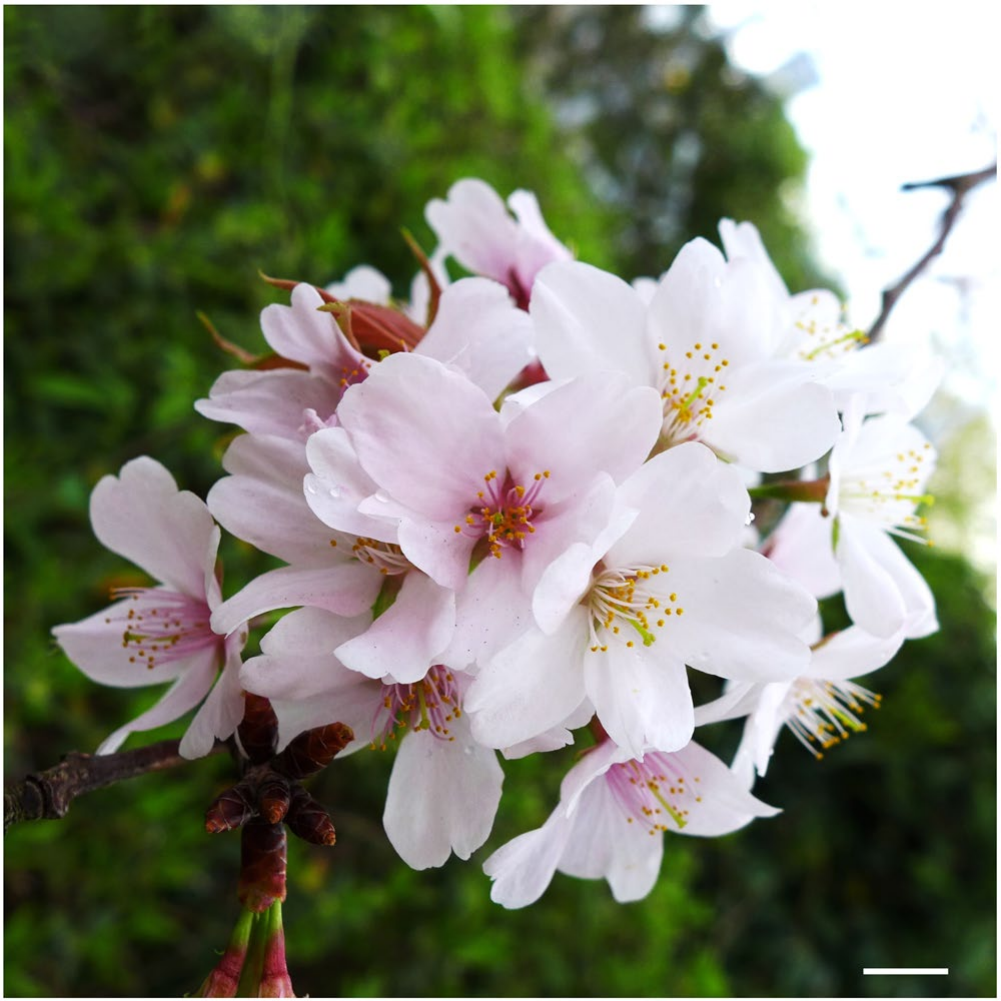
A cherry blossom, *Prunus x yedoensis* cv. Somei-Yoshino, Japanese flowering cherry. The photo was taken in the campus of Hamamatsu University School of Medicine (Hamamatsu, Japan). Scale bar, 1 cm.

*Prunus × yedoensis* cv. Somei-Yoshino is a hybrid cherry blossom formed by a cross between *Prunus speciosa* (Oshima zakura) and *Prunus pendula* f. *ascendens* (Edo higan) (Fig. 1). Somei-Yoshino is the most popular and widely planted cherry tree in Japan and, under good conditions, it grows to a height of 15 meters in 50 years. These cherry trees have inherited two major properties: a large wheel-shaped flower from *Prunus speciosa*, and flowers that bloom before leaves form from *Prunus pendula* f. *ascendens*, resulting in one of the best cherry trees for viewing. For the present experiments, we chose petals without any cracks or bruises on the surface (see Methods).

**Figure 2:**
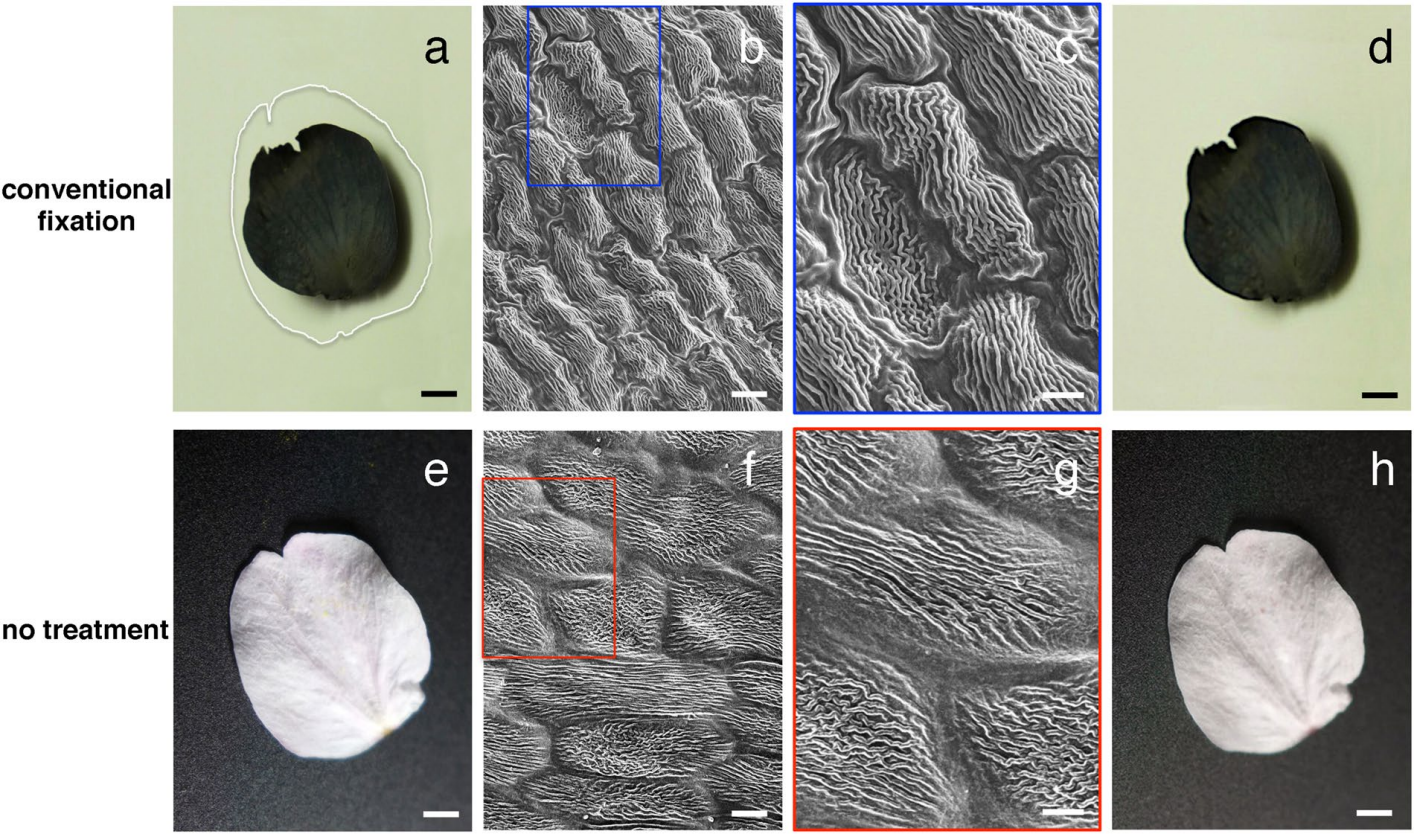
Observations of petals of a cherry blossom by light microscopy (LM) (**a**,**d**,**e**,**h**) and scanning electron microscopy (SEM) (**b**,**c**,**f**,**g**). Comparison of LM images of the petals before (**a**,**e**) and after the SEM observations (**d**,**h**). The white line surrounding the darkened petal in (**a**) indicates the original shape of the petal before conventional fixation and sample preparation. Scanning electron micrographs compare specimens prepared by conventional methods for SEM (**b**,**c**) and specimens untreated (**f**,**g**). The rectangles in (**b**,**f**) indicate the position of images magnified in (**c**,**g**). Scale bars, 2 mm (**a**,**d**,**e**,**h**), 10 μm (**b**,**f**), 5 μm (**c**,**g**).

Figure 2a–d shows typical results of FE-SEM imaging of cherry blossom petals prepared by conventional fixation methods. Under these conditions, shrinkage of specimens was inevitable due to dehydration (Fig. 2a). By comparison, we introduced cherry blossom petals directly into the SEM to see how they changed under high vacuum (10^−3^–10^−6^ Pa). Surprisingly, without any pre-treatment, the overall morphology of living specimens was well preserved in the SEM, and samples showed no electrostatic charging (Fig. 2f,g), which prevents satisfactory imaging^12^. We found that the size of cells in living petals remained large, when compared to conventionally fixed specimens (Fig. 2b,c versus Fig. 2f,g). The petals also showed no observable change in appearance before (Fig. 2e) and after 20 minutes SEM observations (Fig. 2h), suggesting that these untreated specimens remained “wet” during the SEM observation. This unexpected finding led to the hypothesis that a barrier to gas and/or liquid loss is formed, which is similar to the “NanoSuit” formed on the surface of animals in the SEM.

The basic principal of the NanoSuit is to use the ECS covering some organisms or to add a substance mimicking the ECS and to polymerize these substances by electron beam or plasma irradiation^9,13^. To test whether a NanoSuit is formed on living petals, samples were placed in the SEM observation chamber for 20 minutes at an identical vacuum level, but without concurrent electron beam radiation. The results in Table 1 show that living petals, which were not irradiated with an electron beam, lost 20% of their weight, whereas specimens irradiated with electron beam for 20 minutes showed only 5% weight loss. Specimens prepared by conventional methods did not lose weight during SEM observations, because they had already lost almost 80% of their weight during sample preparation (data not shown; cf. Fig. 2a,d). These results suggest that, with the assistance of energy from an electron beam, living petals possess the ability to tolerate high vacuum environments, presumably because substances on the outer surface of petals turn into a NanoSuit similar to that formed by animal ECS.

**Table 1:** Weight loss of unfixed living petals after SEM observation.

Weight loss in samples of petal (cherry blossom) treated with ethanol or chloroform, and exposed to high vacuum in an SEM for 20 minutes (*N* = 5)	
no treatment (not irradiated)	20.1 ± 5.1%
no treatment (irradiated with electron beam)	5.3 ± 2.6%
treated with ethanol (irradiated with electron beam)	7.7 ± 5.5%
treated with chloroform (irradiated with electron beam)	73.9 ± 10.3%

### Wax-based surface substances turn into a NanoSuit when exposed to an electron beam

**Figure 3:**
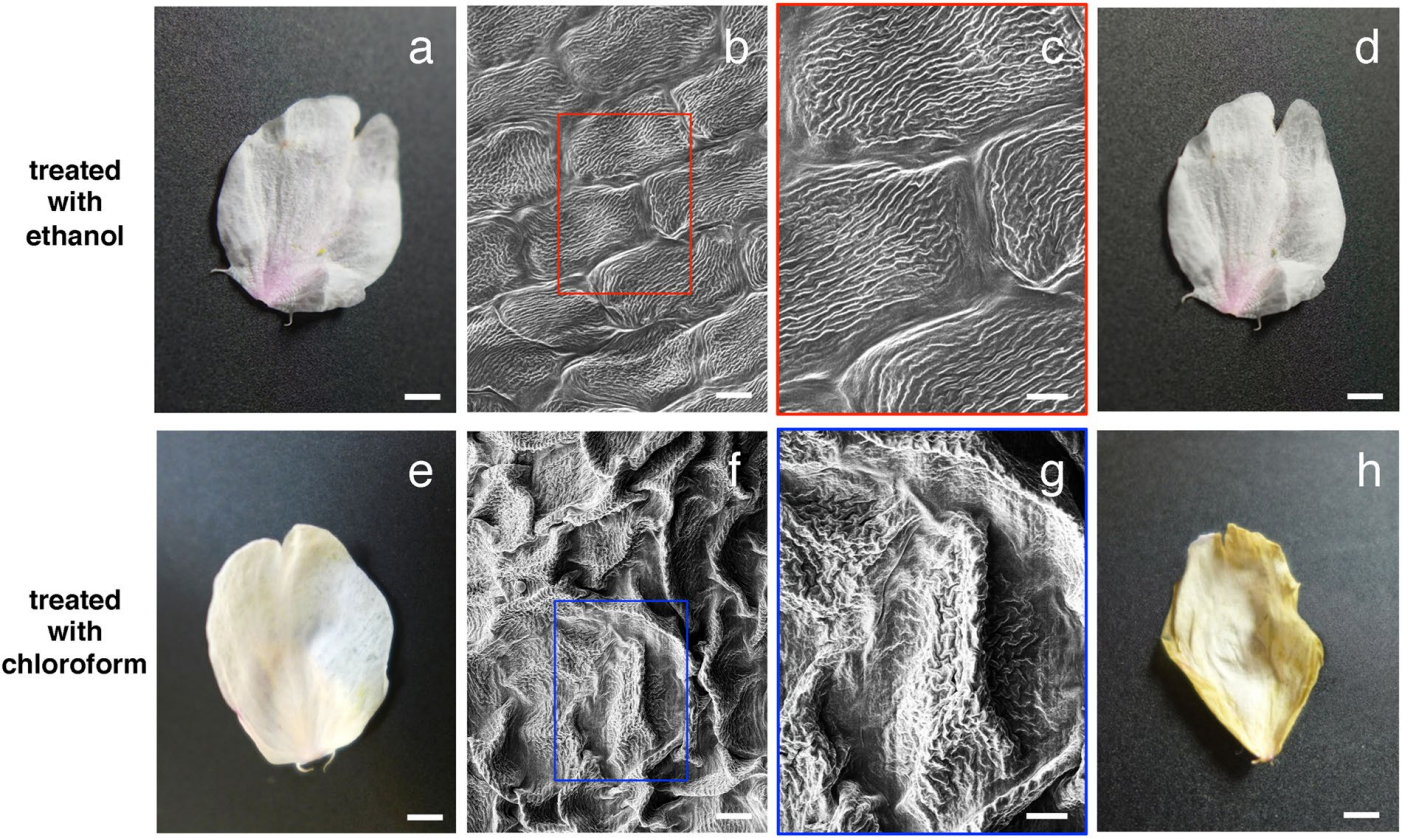
Comparison of LM and SEM images of petals treated with ethanol (**a**–**d**) and chloroform (**e**–**h**). LM images (**a**,**d**,**e**,**h**) and SEM images (**b**,**c**,**f**,**g**). Comparison of LM images of the petals before (**a**,**e**) and after the SEM observations (**d**,**h**). The rectangles in (**b**,**f**) indicate the position of images magnified in (**c**,**g**). Scale bars, 2 mm (**a**,**d**,**e**,**h**), 10 μm (**b**,**f**), 5 μm (**c**,**g**).

**Figure 4:**
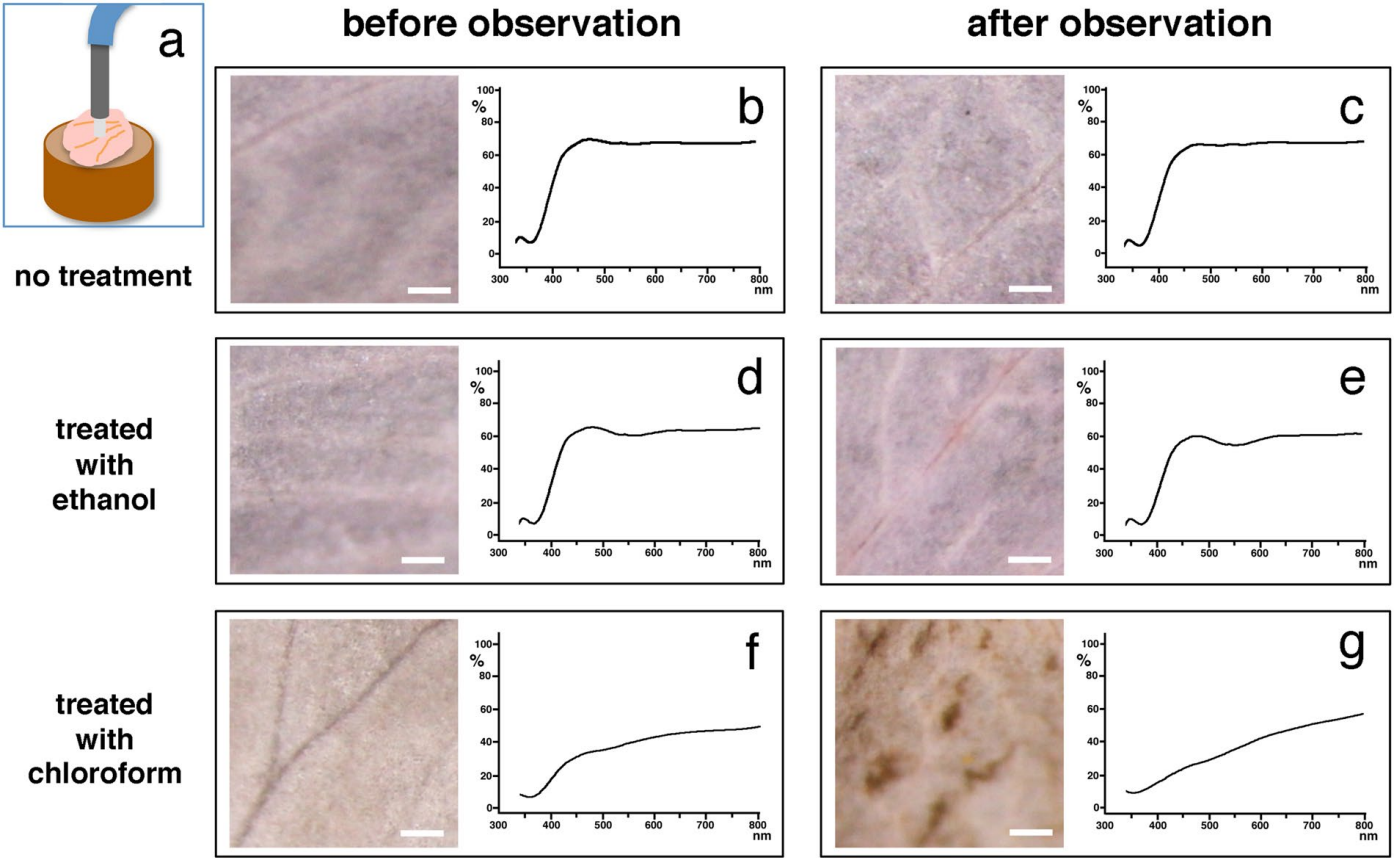
A schematic drawing of the measurement for reflection spectrum (**a**). Comparison of the reflection spectra in petals before (**b**,**d**,**f**) and after the SEM observations (**c**,**e**,**g**). The petals untreated (**b**,**c**), treated with ethanol (**d**,**e**) and chloroform (**f**,**g**). Corresponding LM images are shown in each box. Scale bars, 0.1 mm.

Plant wax forms a diffusion barrier covering the outer surfaces of plants^3,4,7^. To further investigate the relationship between plant surface substances and the NanoSuit, we tested whether they could be extracted with organic solvents (see Methods). After the treatments, we introduced the specimens into the SEM to see how the structures changed during the observations. When the petals were treated with ethanol, the specimens showed no morphological change compared to controls based on stereo dissecting microscopic (Fig. 3a,d) and electron microscopic observations (Fig. 3b,c). On the other hand, in the specimens treated with chloroform, cells in petals shrank and collapsed, and showed electrostatic charging during SEM observation (Fig. 3f,g), leading to an overall change in morphology after the observations (Fig. 3e,h). Figure 4 shows the reflection spectra of these samples. Both untreated specimens (Fig. 4b,c) and specimens treated with ethanol (Fig. 4d,e) showed little difference in the spectra, whereas the reflection spectrum of specimens treated with chloroform (Fig. 4f,g) changed markedly. Specimens treated with ethanol showed only 7.7% decrease in weight whereas specimens treated with chloroform showed a 74% decrease in weight after 20 minutes of SEM observations (Table 1). These results suggest that substances on the surface of petals behaved like wax and probably played a significant role as a barrier to reduce the effects of high vacuum.

**Figure 5:**
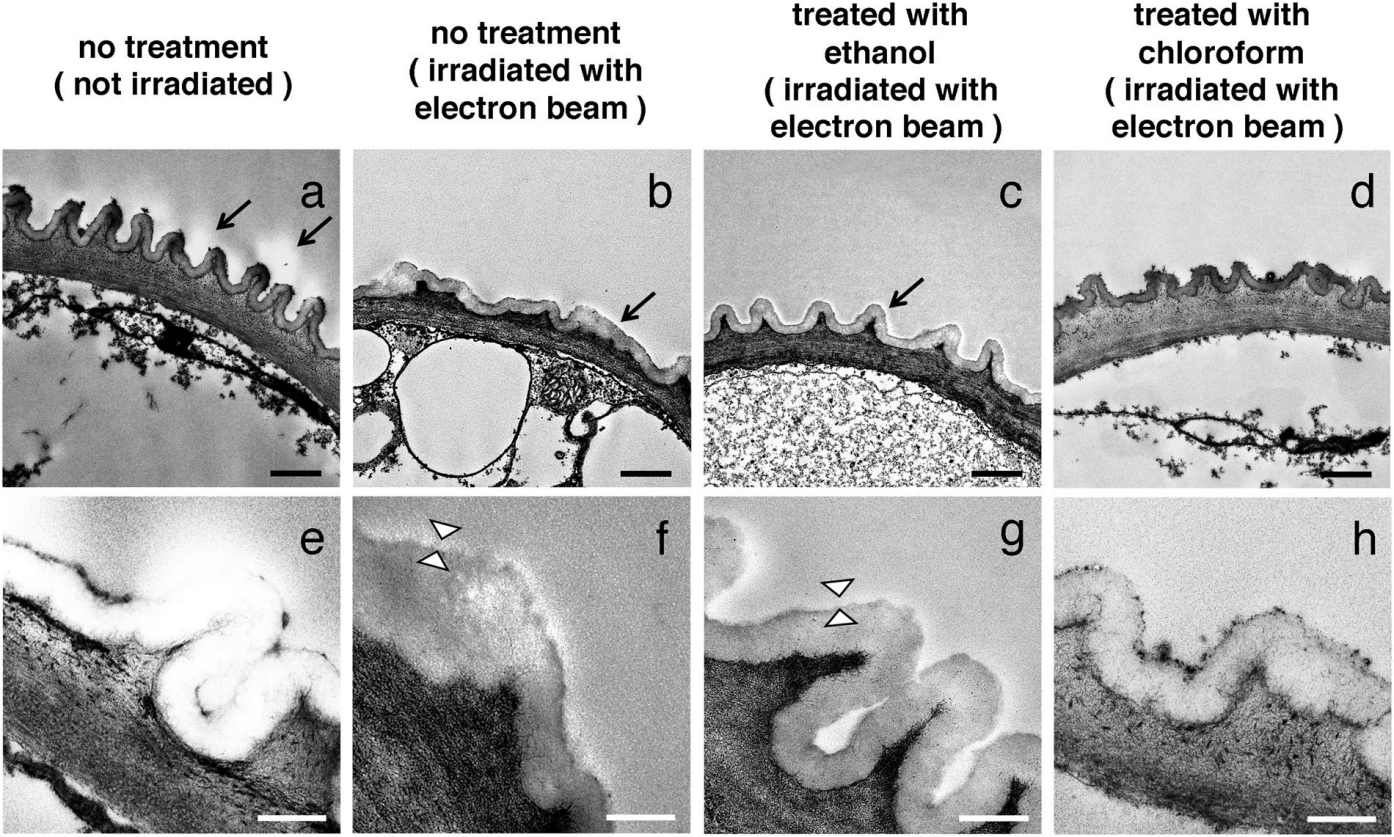
Imaging the plant NanoSuit. TEM mages of cross sections through petals, without electron beam irradiation (**a**,**e**), with electron beam irradiation (**b**,**f**), treated with ethanol and electron beam irradiation (**c**,**g**) and treated with chloroform and electron beam irradiation (**d**,**h**). All specimens were then fixed and sectioned for TEM. The arrows in (**a**–**c**) indicate the position of the surface material covering petals (white layers). The layer between the arrowheads in (**f**,**g**) indicates the newly formed NanoSuit. Scale bars, 1 μm (**a**–**d**), 200 nm (**e**–**h**).

To identify this wax-based surface substance morphologically, we fixed and stained petals of cherry blossom for transmission electron microscopy (TEM) (Fig. 5). Cross-sections showed that two control specimens, irradiated or not irradiated with the electron beam, and specimens treated with ethanol and irradiated with the electron beam, had an extra layer of material covering their surface (white layers indicated by arrows in Fig. 5a–c). Since no such substance was detected in specimens, which were treated with chloroform and irradiated with the electron beam (Fig. 5d,h), we conclude that this substance is the wax-based surface substance on the petals. Observations at higher magnification revealed that, both in control specimens irradiated with the electron beam and in specimens treated with ethanol and irradiated, the surface layer became thinner (less than 50 nm thickness) (Fig. 5f,g), suggesting that the wax-based material was polymerized by the electron beam and that a NanoSuit was formed.

**Figure 6:**
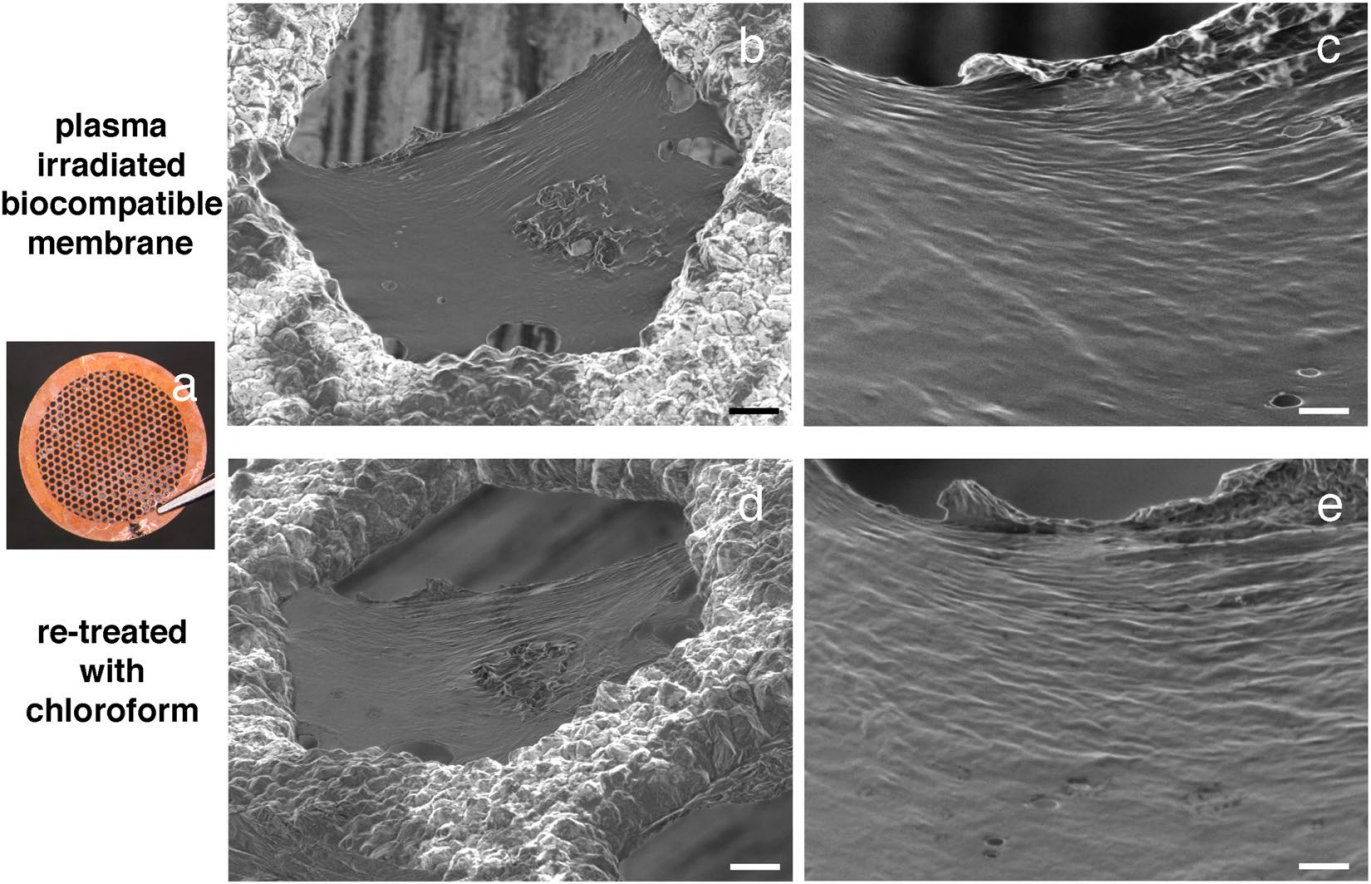
Images of newly synthesized membranes from the natural surface material of petals following plasma polymerization. The membrane was polymerized over each hole of the 150 A copper grid mesh (**a**). The membrane surface as observed by SEM (**b**,**c**). The polymerized membrane was insoluble in chloroform and its surface could be observed by SEM (**d**,**e**). Scale bars, 10 µm (**b**,**d**), 2 µm (**c**,**e**).

Finally, we tested whether the wax-based surface material on petals could be polymerized into a biocompatible membrane (see Methods). The surface material extracted with chloroform was dried on a copper grid mesh, and irradiated by plasma (Fig. 6a). This led to the formation of a membranous structure (Fig. 6b,c), which was insoluble in chloroform, clearly showing the effects of plasma polymerization (Fig. 6d,e).

In summary, electron beam irradiation leads to the formation of a thin layer on the surface of petals which protects them from desiccation under the high vacuum in the SEM. This layer is equivalent to the NanoSuit, which has been shown to protect living animal tissue in the SEM^9,13^.

## Discussion

We have previously found that, with the assistance of a polymerized thin film, called a “NanoSuit”, some small animals can tolerate high vacuums, because the NanoSuit acts as a barrier to gas and liquid loss from the organism^9–13^. To understand how organisms can avoid desiccation in a high vacuum would be a major advance in an attempt to more fully understand the role of the surface barrier in protecting the organism. Until now little was known about such a protection system in plants. In the present investigations, we used the petals of cherry blossom as experimental specimens and examined their response to high vacuum environments. Since the basic concept of the NanoSuit is to use the ECS covering some animals, we focused on the natural substances covering the surface of plants.

Plant cuticular wax forms a diffusion barrier covering the outer surface of plant tissues and plays a role in preventing non-stomatal water loss from the plant surface^3,4,7^. Moreover, it has also been shown that increased amounts of cuticular wax are associated with improved drought tolerance in several grains^14^. The present study has demonstrated that wax-based surface materials on petals turn into a NanoSuit following exposure to an electron beam (Figs 2, 3, 5). In the presence of the plant NanoSuit, specimens showed only 5% weight loss in the SEM (Table 1). By comparison, specimens treated with chloroform, which did not form a NanoSuit, showed a 74% decrease in weight after SEM observation (Table 1). Based on the results presented here, we suggest that, while wax-based surface materials can prevent water loss from the plant surface, conversion of this material into a NanoSuit by electron beam irradiation can significantly increase the barrier ability.

We have reported that various biocompatible membranes can be fabricated by plasma polymerization from surfactants, water-soluble polymers, monosaccharides, polysaccharides, lipids, amino acids, and ionic liquids^10^. Moreover, the SSE solution, which consists of glycerin and electrolytes, interacts with cells to form a much thinner NanoSuit with stronger barrier ability^13^. In plants, the chemical basis and extent of the differentiation of waxes varies between species^1^. Although specific components of wax have not been defined in our experiments, their removal by chloroform treatment prevented NanoSuit formation. Conversely, the material extracted with chloroform could be polymerized into a membranous structure by plasma irradiation (Fig. 6), suggesting that wax is a new candidate for forming a NanoSuit.

To protect themselves from the external environment, multicellular organisms usually possess their own ECS; e.g. biofilms of microbes, ECS of insect larva, surfactants of the vertebrate lung. The present report has revealed that wax-based plant surface materials work in a similar way to the animal ECS. Therefore, future studies to investigate the property of petals in other species and/or other plant organs need to examine the wax-based surface materials. This new concept will improve the depth of our understanding not only in bio-related fields but in a range of other scientific disciplines as well.

## Methods

### Experimental organisms

Most samples in the present study came from the Japanese flowering cherry (*Prunus x yedoensis* cv. Somei-Yoshino), the most popular ornamental tree species in Japan. The petals were collected in the campus of Hamamatsu University School of Medicine (137°72′E, 34°70′N) during the day in the blooming seasons from 2015 to 2017.

### Treatment with solvents, and weight loss experiment

To remove excess solution remaining on the surface of the petals collected under natural conditions, the specimens were exposed to low vacuum (ca. 10 Pa) for 1 min 30 sec, and then weighed. This weight was defined as the starting weight of the petal. If a petal had bruises on the surface, it was rejected. Only healthy petals were selected for experiments. Except for the control experiment, healthy petals of the blooming cherry were wiped gently with filter paper containing ethanol or chloroform and then weighed. After FE-SEM observations, specimens were weighed again to determine the water loss. The difference before and after observations was indicated as a percentage. Statistical analysis was done with the *t-*test, significance level set at *P* < 0.01.

### Sample preparation for the FE-SEM to observe living petals

Healthy control petals and petals treated with ethanol or chloroform were introduced into the SEM directly without any further treatments such as chemical fixation, dehydration and ultrathin coating of electrically conducting materials.

### Preparation for standard scanning and transmission electron microscopy

For conventional SEM observations, petals of the flowering cherry were prefixed with 2% glutaraldehyde in 0.1 M cacodylate buffer (pH7.4) and postfixed in 1% OsO_4_ in the same buffer. The specimens were then dehydrated, freeze dried (JFD300, JEOL), and ultra-thin coated with OsO_4_ (PMC-5000, Meiwa). For transmission electron microscopy (TEM), specimens were prefixed in 2% glutaraldehyde in 0.1 M cacodylate buffer (pH7.4), and then postfixed in 1% OsO_4_ in the same buffer. The dehydrated specimens were embedded in an Epon-Araldite mixture. Ultra-thin sections (approximately 70 nm) were cut (ULTRACAT OmU_4_, REICHERT-JUNG) vertical to the surface and were stained with 2% uranyl acetate followed by 0.4% lead citrate for 5 min each.

### Microscopy

Field emission scanning electron microscopy was carried out with a JEM-7100F (JEOL) and/or a Hitachi S-4800 instruments operated at acceleration voltages of 1.0 kV. The vacuum level of the observation chamber was 10^−3^~10^−6^ Pa. The detector for secondary electrons was a signal from lower detector. Other details are as follows; working distance: 8 mm, aperture size: φ 100 µm, scan speed: each beam is 10–15 frames/sec. Transmission electron microscopy (TEM) observations were carried out using a JEM-1220 (JEOL) at an acceleration voltage of 120 kV.

### Measurements of the reflection spectrum

The spectral reflectance of petals was measured with a USB2000 spectrophotometer (Ocean Optics, Inc., Ostfildern, Germany) and illumination was provided by a DH-2000-BAL light-source (Ocean Optics, Inc., Ostfildern, Germany), both connected via coaxial fiber cable (QR400–7-UV-VIS, Ocean Optics, Inc., Dunedin, FL, USA). Reflectance was measured relative to a standard white reference tile (Diffuse Reflectance Standard, Spectralon®, labsphere). All reflectance measurements were taken in a constant angle of 90° towards the upper surface of each petal, and the distance between the probe tip and the sample surface was kept constant (5 mm) (cf. Fig. 4a).

### Plasma polymerization for fabricating biocompatible membranes

Preparation of insoluble plasma-irradiated membranes was performed as described by Takaku *et al*.^9^. Briefly, the metal-emitter from a standard ion-sputtering device (MSP-20-UM,VACUUM DEVICE) was removed, so that the plasma ions produced within the chamber were based on the remaining air-derived gas molecules. To make a free-standing membrane, the surface material on petals was extracted with chloroform and spread on a copper grid mesh (Cu150P, Okenshoji Co.) (cf. Fig. 6a). After the chloroform evaporated, the extracted materials remaining on the grid mesh were polymerized by plasma irradiation. Specimens were irradiated at room temperature with plasma for 10 min at ca. 30 mA (+0.56 kV) under a vacuum level of ca. 10 Pa. The thickness of the membrane could be controlled both by the duration of irradiation time and the concentration of the solution.
